# Molecular characterization of fluoroquinolone resistance in invasive clinical isolates of *Streptococcus pneumoniae* susceptible to delafloxacin

**DOI:** 10.1093/jac/dkaf308

**Published:** 2025-08-21

**Authors:** Emilia Cercenado, Mercedes Marín, Manuel Iglesias, Laura Jiménez, Marta Pérez-Abeledo, Juan Carlos Sanz

**Affiliations:** Servicio de Microbiología Clínica y Enfermedades Infecciosas, Hospital General, Universitario Gregorio Marañón, Madrid, Spain; Instituto de Investigación Sanitaria Hospital Gregorio Marañón, Madrid, Spain; Medicine Department, School of Medicine, Universidad Complutense, Madrid, Spain; CIBER Enfermedades Respiratorias-CIBERES (CB06/06/0058), Madrid, Spain; Servicio de Microbiología Clínica y Enfermedades Infecciosas, Hospital General, Universitario Gregorio Marañón, Madrid, Spain; Instituto de Investigación Sanitaria Hospital Gregorio Marañón, Madrid, Spain; Medicine Department, School of Medicine, Universidad Complutense, Madrid, Spain; CIBER Enfermedades Respiratorias-CIBERES (CB06/06/0058), Madrid, Spain; Medical Department, Menarini, Madrid, Spain; Servicio de Microbiología Clínica y Enfermedades Infecciosas, Hospital General, Universitario Gregorio Marañón, Madrid, Spain; Unidad de Microbiología Clínica, Laboratorio Regional de Salud Pública de la Comunidad de Madrid, Madrid, Spain; Unidad de Microbiología Clínica, Laboratorio Regional de Salud Pública de la Comunidad de Madrid, Madrid, Spain

## Abstract

**Background:**

Delafloxacin is a dual-targeting fluoroquinolone against topoisomerase IV and DNA gyrase that could decrease resistance selection by diminishing the likelihood of multiple mutational events in both enzymes.

**Objectives:**

To determine the activity of delafloxacin against invasive *Streptococcus pneumoniae* isolates resistant to levofloxacin (LEV-R), compare delafloxacin MICs for LEV-R isolates with those of susceptible strains, and analyse mutations in QRDRs.

**Methods:**

A total of 130 *S. pneumoniae* isolates (2014–20) were studied. The isolates were distributed according to levofloxacin MICs: high-level LEV-R (*n* = 46; MIC > 32 mg/L), low-level LEV-R (*n* = 36; MIC range 3–12 mg/L) and susceptible (LEV-S; *n* = 48; MIC ≤2 mg/L). We considered delafloxacin-resistant to be MIC ≥ 0.12 mg/L (EUCAST epidemiological cut-off). MICs were determined by gradient diffusion (control strain *S. pneumoniae* ATCC 49619). All isolates were subjected to PCR and sequencing of *parC*, *parE*, *gyrA* and *gyrB* genes.

**Results:**

All LEV-S isolates showed delafloxacin MICs of ≤0.008 mg/L, and did not show mutations in QRDRs. Isolates with levofloxacin MICs of 3–12 mg/L showed delafloxacin MICs of <0.12 mg/L, with 3 (8.3%) presenting mutations in *gyrA*, and 11 (30.6%) in *parC* previously related to resistance. Isolates with levofloxacin MICs of >32 mg/L showed two to four mutations in QRDRs and 11 (24%) were delafloxacin resistant, presenting at least two mutations in *gyrA*S81F/L/V *+* *parC*S79F; four accumulated three mutations, and two showed four mutations in QRDRs.

**Conclusions:**

Among LEV-R pneumococci, 71 (87%) were susceptible to delafloxacin, indicating that it maintains its activity despite the presence of mutations in *gyrA* *+* *parC* that lead to high-level resistance to levofloxacin.

## Introduction

Conjugate vaccines have been an effective measure for the control of pneumococcal infection. However, replacement by non-vaccine serotypes is worrisome.^1^ In this context, antibiotic resistance in *Streptococcus pneumoniae* represents a health warning^2^ affecting both bacteriostatic and bactericidal agents.^3^ Within the bactericidal agents are the quinolones, the mechanism of action of which affects the synthesis of bacterial DNA. In general, resistance to this group of antibiotics is related to chromosomal mutations in the drug targets (DNA gyrase and topoisomerase), a reduction in porin synthesis or efflux pumps.^4^

Delafloxacin [1-(6-amino-3,5-difluoropyridin-2-yl)-8-chloro-6-fluoro-7-(3-hydroxyazetidin-1-yl)-4-oxo-1,4-dihydroquinoline-3-carboxylic acid] is a new agent having structural differences with other fluoroquinolones that grant it greater antibacterial activity.^4,5^ Against pneumococci it has a dual mechanism of action, exhibiting high affinity for both topoisomerase IV (ParC_2_ParE_2_) and DNA gyrase (GyrA_2_GyrB_2_).^6^ The fluoroquinolones currently in clinical use generally differ in their ability to inhibit the two target enzymes, and mutations in only one of these enzymes lead to an 8–16-fold increase in MIC values.^7,8^ High resistance of pneumococci to levofloxacin suggests the accumulation of mutations affecting both ParC and GyrA or ParE and GyrA.^9–11^ In a previous study, we evaluated the activity of delafloxacin against 173 invasive high-level levofloxacin-resistant pneumococcal isolates (MIC > 32 mg/L), which showed high activity, with MIC_50_ and MIC_90_ values of 0.064 and 0.12 mg/L, respectively (range: ≤0.002 to 0.5 mg/L), with only one isolate (serotype 9V) showing a delafloxacin MIC of 0.5 mg/L.^12^

We aimed to determine the activity of delafloxacin against invasive levofloxacin-resistant pneumococcal strains, to compare delafloxacin MICs against high- and low-level levofloxacin-resistant and susceptible strains, to understand the role of potential mutations in *parC*, *gyrA* and *parE* genes in its activity, and to perform a structural analysis of inhibition complexes to illustrate the potential role of the different mutations.

## Methods

### Bacterial isolates

A total of 130 *S. pneumoniae* strains causing invasive disease, isolated between 2014 and 2020 from usually sterile clinical samples, were studied. The strains were identified based on phenotypic characteristics. Capsular serotypes were determined by Pneumotest-Latex and by Quellung reaction (Statens Serum Institut, Copenhagen, Denmark). The isolates were distributed in three groups: (i) 46 isolates with high-level resistance to levofloxacin [MIC > 32 mg/L (all from adults aged 43–97 years; 44 from blood culture, 1 from pleural fluid, 1 from ascitic fluid)]; (ii) 36 with low-level levofloxacin resistance [MIC 3–12 mg/L; (3 from children aged 5 months to 5 years, 33 from adults aged 22–93 years; 33 from blood culture, 2 from pleural fluid, 1 from CSF)]; (iii) 48 ‘susceptible with increased exposure’ isolates, referred to as susceptible from now on [MIC ≤ 2 mg/L; (3 from children aged 7–11 months, 45 from adults aged 24–94 years; 47 from blood culture, 1 from CSF)]. MIC interpretations were in accordance with EUCAST^13^ (EUCAST v.15; www.eucast.org). Strains were classified and selected according to different pneumococcal serotypes (Table 1).

**Table 1.** Distribution of *S. pneumoniae* isolates according to serotypes and levofloxacin MICs

Serotype	Levofloxacin MIC (mg/L)			Total
	≥ 32	3–12	≤2	
8	16	3	12	31
9V	10	0	16	26
14	10	0	5	15
15A	7	1	7	15
4	0	7	0	7
19A	1	2	2	5
9N	0	5	0	5
11A	1	3	2	6
31	1	2	1	4
Serogroup 33^a^ (non-subtypeable)	0	0	2	2
35F	0	1	1	2
6C	0	2	0	2
17A	0	2	0	2
24F	0	1	0	1
3	0	1	0	1
Serogroup 7^a^ (non-subtypeable)	0	1	0	1
10A	0	1	0	1
12B	0	1	0	1
12F	0	1	0	1
15C	0	1	0	1
23A	0	1	0	1
Total	46	36	48	130

^a^These strains could not be typed at serotype level.

### Antimicrobial susceptibility testing

Delafloxacin and levofloxacin MICs were determined by the gradient diffusion method [Liofilchem, Italy (delafloxacin); ETEST^®^ bioMérieux, France (levofloxacin)]. *S. pneumoniae* ATCC 49619 was used as a control strain.

Following EUCAST guidelines,^13^  *S. pneumoniae* isolates with a levofloxacin MIC of >2 mg/L were considered to be levofloxacin resistant. Since a breakpoint for delafloxacin and *S. pneumoniae* has not yet been established by EUCAST, we considered isolates to be resistant when the MIC was ≥0.12 mg/L, since this is the epidemiological cut-off (ECOFF) value established by EUCAST^13^ (https://mic.eucast.org/search/).

### PCR of QRDRs and DNA sequence analysis

All 130 isolates were subjected to PCR and amplicon Sanger sequencing of the *gyrA*, *gyrB*, *parC* and *parE* genes as previously described.^9,14^ To amplify the *gyrA* and *parE* genes, we used the oligonucleotides gyrA44 and gyrA170, and parE398 and parE483, respectively.^14^ To amplify the *gyrB* and *parC* genes, we used the oligonucleotides gyrB376 and gyrB512, and parC50 and parC152, respectively.^9^ All sequences obtained were compared with the control strain WT *S. pneumoniae* ATCC 49619 but also with sequences of each complete gene deposited in GenBank: *gyrA* sequence LN831051.1, *gyrB* sequence DQ175243.1, *parC* sequence AAF85902.1 and *parE* UYIP010000001.1, using BioEdit software version 7.2. The role of the different mutations in *gyrA*, *parC*, and *parE* in pneumococcal resistance to fluoroquinolones was assigned according to previous studies.^6,15^ In these studies, mutations S81F, S81V, E85G, S83F and D87N in *gyrA*, S79F, S79Y, S80P, D83E and D83N in *parC*, and D435N in *parE* were considered to be highly related to resistance (Table 2).

**Table 2.** QRDR mutations related to resistance to fluoroquinolones in *S. pneumoniae* (according to de la Campa *et al.*^6,15^)

Genes	Mutations conferring resistance	Genes	Mutations that do not confer resistance
*gyrA*	S81V	*gyrA*	G79A
	S81F		V101I^a^
	E85G		
	S83F		
	D87N		
*parC*	S79F	*parC*	D78N
	S79Y		K137N
	S80P		N91D
	D83E		
	D83N		
*parE*	D435N	*parE*	I476F
			I460V

^a^Usually found with S81F; role in resistance alone is unclear.

### Structural analysis of inhibition complexes

The coordinates of *S. pneumoniae* gyrase (PDB: 4Z2E) and topoisomerase (PDB: 4KOE) were used as the receptor structures. Hydrogen atoms and partial charges at pH 7.0 were added using the pdb2pqr software.^16^ The coordinates of the ligand, delafloxacin, were obtained by atom-by-atom modification of the trovafloxacin ligand using the *sculpt* option in PyMOL, followed by energy minimization *in vacuo* with 2500 cycles of conjugate gradient, and 500 cycles of steepest descent, using the AMBER12 package.^17^

**Figure 1.**
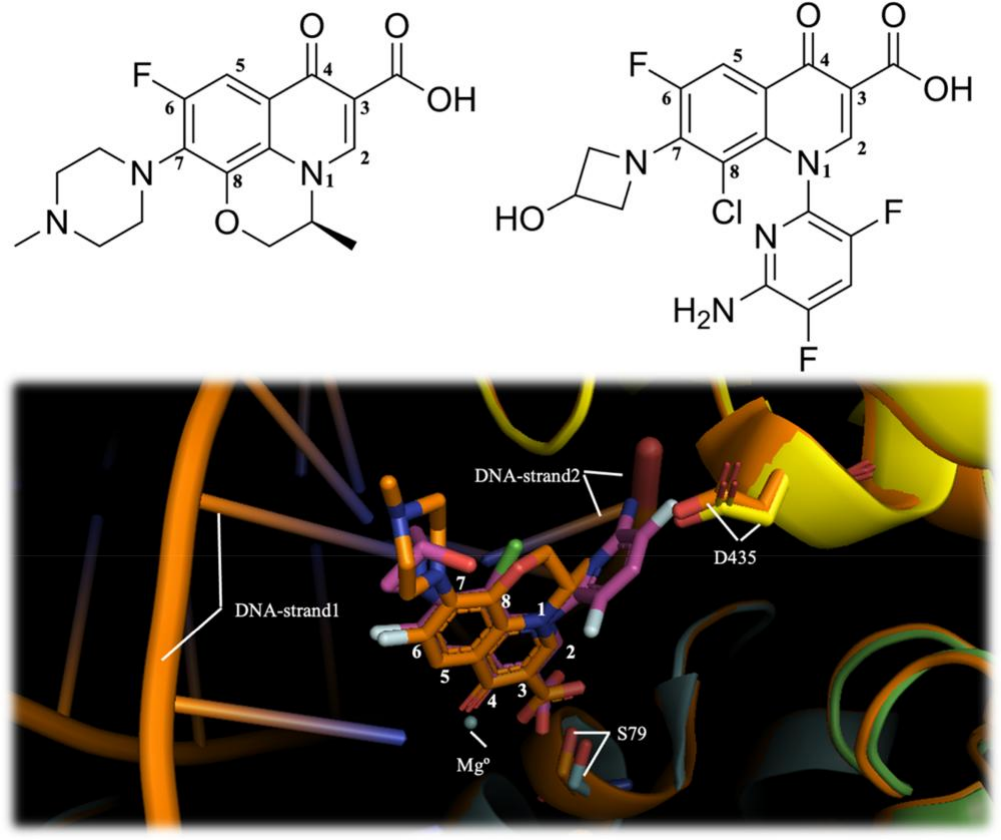
Top: chemical structures of levofloxacin (left) and delafloxacin (right). Bottom: imposed structures of delafloxacin (purple) and levofloxacin (orange) in the *S. pneumoniae* topoisomerase complex.

To understand the role of different mutations in delafloxacin sensitivity, the interactions of the crystallographic structures of levofloxacin complexes with *S. pneumoniae* gyrase (PDB: 4Z2D) and topoisomerase (PDB: 3RAE) were analysed. This was done by structural superposition with the corresponding modelled delafloxacin–gyrase and delafloxacin–topoisomerase complexes (Figure 1).

### Statistical analysis

The comparison of qualitative variables was performed using the Fisher exact test. Results were considered significant when *P* ≤ 0.05.

### Ethics

This study was approved by the Ethics Committee for Research Involving Drugs of the University Hospital Gregorio Marañón, Madrid, Spain. Code number: MICRO.HGUGM.2021-015.

## Results

### Serotype distribution and susceptibility analysis

**Figure 2.**
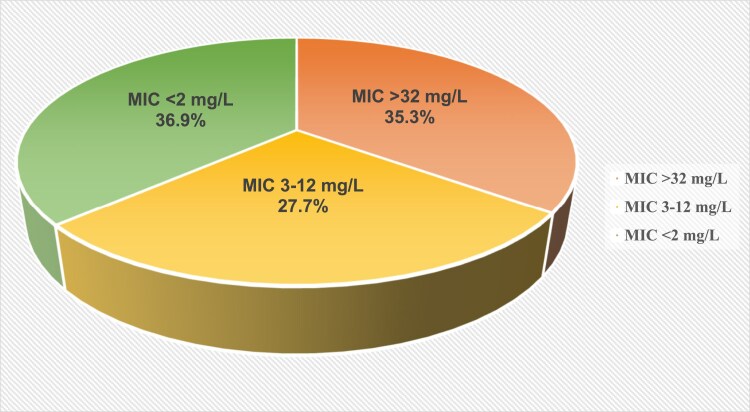
Distribution of the 130 isolates analysed according to levofloxacin MICs.

The distribution of levofloxacin-susceptible (36.9%), low-level resistant (27.7%) and high-level resistant (35.3%) isolates is shown in Figure 2.

The distribution of isolates according to serotypes and delafloxacin MICs (mg/L) is shown in Table 3. The 11 isolates with delafloxacin MICs of ≥0.12 mg/L corresponded to serotypes 9V (*n* = 4), 8 (*n* = 3), 15A (*n* = 2), 14 (*n* = 1) and 19A (*n* = 1).

**Table 3.** Distribution of *S. pneumoniae* isolates according to serotypes and delafloxacin MICs

Serotype	Delafloxacin MIC (mg/L)						Total
	<0.064	0.064	0.094	0.12	0.19	0.25–0.5	
8	18	3	7	3			31
9V	19	3		1	1	2	26
14	10	3	1	1			15
15A	12	1		1		1	15
4	7						7
19A	4				1		5
9N	5						5
11A	5	1					6
31	3		1				4
Serogroup 33^a^ (non-subtypeable)	2						2
35F	2						2
6C	2						2
17A	2						2
24F	1						1
3	1						1
Serogroup 7^a^ (non-subtypeable)	1						1
10A	1						1
12B	1						1
12F	1						1
15C	1						1
23A	1						1
Total	99	11	9	6	2	3	130

^a^These strains could not be typed at serotype level

All 48 isolates (100%) susceptible to levofloxacin were susceptible to delafloxacin (MIC of ≤0.008 mg/L), as well as all the isolates with a range of levofloxacin MICs between 3 and 12 mg/L (delafloxacin MICs of ≤0.06 mg/L). Regarding the high-level levofloxacin-resistant isolates, 35 (76.1%) were susceptible to delafloxacin (MIC ≤ 0.06 mg/L) (*P* < 0.00001); the range of delafloxacin MICs for the remaining 11 highly levofloxacin-resistant isolates was 0.12–0.5 mg/L. Results are shown in Table 4.

**Table 4.** Distribution of isolates according to delafloxacin and levofloxacin MICs

Levofloxacin MIC (mg/L)	Delafloxacin MIC (mg/L)		Total
	≥0.12	<0.12	
>32	11	35^a^	46
3–12	0	36	36
<2	0	48	48
Total	11	119	130

^a^ *P* < 0.00001.

### Analysis of QRDR genes

#### Comparison with levofloxacin and delafloxacin MICs

None of the 48 levofloxacin-susceptible isolates (100%) presented mutations in either *gyrA*, *gyrB*, *parC* or *parE* associated with resistance; however, two isolates presented the mutation *gyrB*R477G/C, and 19 the mutation *parC*K137N not associated with resistance (Table 2). All were susceptible to delafloxacin.

Among the 36 isolates with levofloxacin MICs of 3–12 mg/L, 23 (63.9%) did not show mutations in either *gyrA*, *gyrB*, *parC* or *parE* associated with resistance. Only three isolates (8.3%) presented mutations in *gyrA*: one isolate had the mutation *gyrA*S81A and two had the mutation *gyrA*S81F. Regarding *parC* mutations, nine isolates showed one single mutation associated with resistance (S79F, S79Y, D83Y) and two isolates showed *gyrA* *+* *parC* mutations (Table 5). One isolate showed the single mutation *parE*D435N associated with resistance (levofloxacin MIC of 3 mg/L). All were susceptible to delafloxacin with a range of MICs between 0.003 and 0.047 mg/L.

**Table 5.** Mutations in isolates with levofloxacin MICs of 3–12 mg/L

No. of isolates^a^	*gyrA* mut^b^	*parC* mut^b^	*parE* mut^b^	Total mutations^b^	Levofloxacin MIC range (mg/L)	Delafloxacin MIC range (mg/L)
23	—	—	—	0	3–4	0.003–0.03
1	—	—	1	1	3	0.003
9	—	1	—	1	3–12	0.003–0.03
1	1	—	—	1	8	0.047
2	1	1	—	2	12	0.003; 0.012

^a^All isolates: levofloxacin MICs were 3–12 mg/L.

^b^mut: mutations; all mutations associated with resistance.

All 46 isolates (100%; *P* < 0.00001) with levofloxacin MICs of >32 mg/L presented mutations in *gyrA*, either one mutation (28 *gyrA*S81F, 6 *gyrA*S81Y, 4 *gyrA*S81L, 1 *gyrA*S81V) or two mutations (5 *gyrA*S81F + *gyrA*E85K, 2 *gyrA*G79A + *gyrA*S81F). In accordance with Table 2, all these mutations have been associated with fluoroquinolone resistance, with the exception of *gyrA*G79A.

None of the isolates with levofloxacin MICs of >32 mg/L showed mutations in *gyrB*.

Regarding *parC* mutations, 38 (82.6%) presented the mutation *parC*S79F (alone or associated with the mutation *parC*K137N, not associated with resistance).

Eleven isolates showed the mutation *parE*D435N, but this was always associated with mutations in either *gyrA*, *parC* or both.

The majority of isolates (*n* = 102; 78.5%) showed the mutation *parE*I460V, regardless of levofloxacin MICs; however, this mutation is considered a polymorphism that does not affect the activity of the fluoroquinolones.

**Figure 3.**
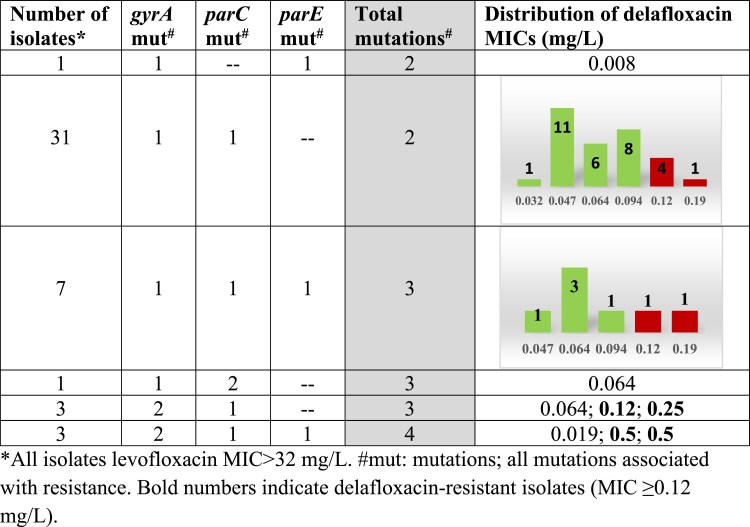
Correlation between accumulation of mutations and delafloxacin MICs among isolates highly resistant to levofloxacin (MIC > 32 mg/L).

The correlation between the accumulation of different mutations (either *gyrA*, *parC* and/or *parE*) and the increase in delafloxacin MICs is shown in Figure 3.

Among isolates highly resistant to levofloxacin, 76.1% (*n* = 35) were susceptible to delafloxacin. The remaining 11 isolates that were resistant to delafloxacin showed at least one mutation in *gyrA* and another in *parC*; however, delafloxacin was also active against 27/32 isolates showing two mutations, against 7/11 isolates showing three mutations, and against 1/3 isolates showing four mutations (Figure 3).

#### Structural analysis of inhibition complexes

**Figure 4.**
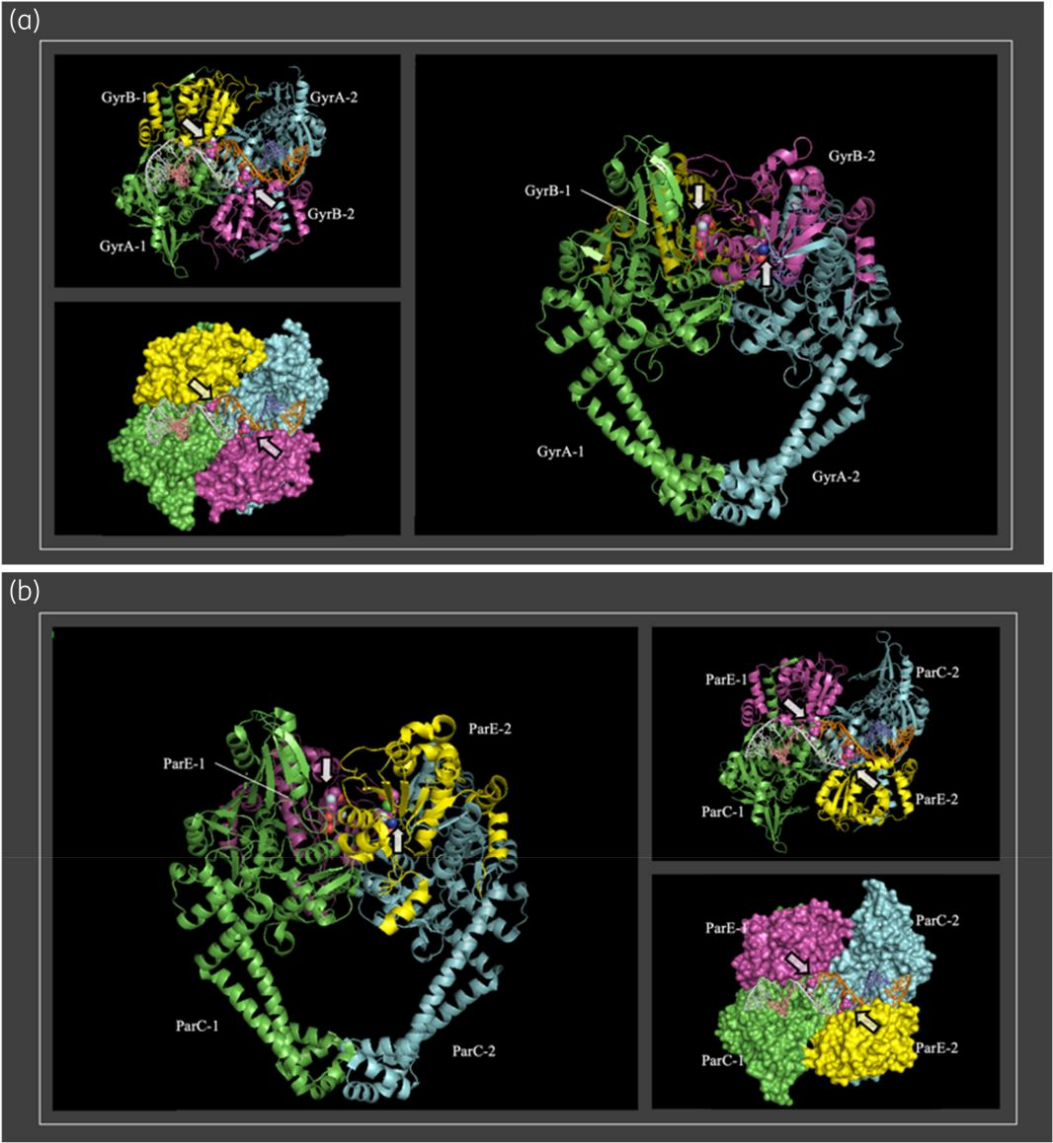
Structural models of gyrase (a) and topoisomerase (b) heterodimers bound to two delafloxacin molecules and an 18 bp DNA strand, serving as the substrate for these enzymes. The sequences of the subunits are identical, so the complexes of delafloxacin and levofloxacin present nearly identical structures (RMSD values of 0110 and 0349 Å for 175 and 194 C_α_ atoms of gyrase and topoisomerase heterodimers, respectively), with minor differences in the quinolone binding site (a). Both models include a cartoon side view of β-strands and α-helices, as well as a top view (90°) represented in cartoon format with surface representations of the subunits in different colours. Arrows indicate the positions of the delafloxacin molecules (represented as spheres).

To illustrate the potential role of the described mutations, Figure 4 shows the generated models of the gyrase and topoisomerase heterodimers binding to two molecules of delafloxacin (highlighted with arrows): two GyrA and two GyrB subunits (Figure 4a), and two ParC and ParE subunits (Figure 4b), respectively. The two delafloxacin molecules (indicated by arrows in Figure 4b) are positioned at the usual binding site, equivalent to that described for other quinolone complexes with these enzymes.^18^ The main differences arise from the substituents at positions 1, 7 and 8 of the two quinolones, which are oriented towards the outside of the cavity near the DNA strands. The core structure, including the quinolone ring with the fluorine atom at position 6, overlaps in the crystallographic complexes of levofloxacin (experimental structure) and the theoretical model of delafloxacin (Figures 1 and 4). It is shown that some of the residues involved in the binding of the fluoroquinolones to their targets are the main mutations that accumulate and increase the MIC values until resistance is generated.

**Figure 5.**
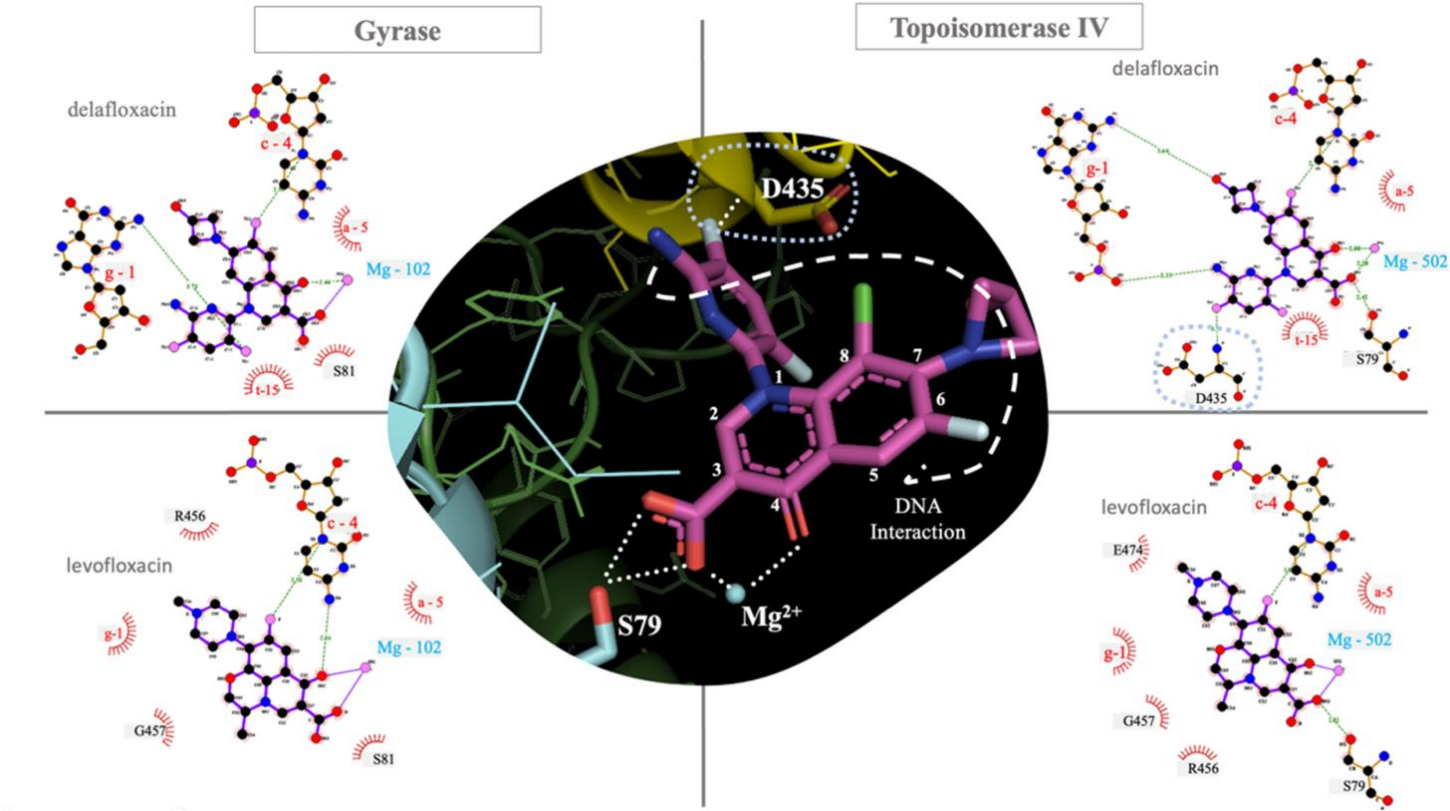
Comparison of the structures of delafloxacin (top) and levofloxacin (bottom) complexes with gyrase (left) and topoisomerase (right) of *S. pneumoniae*. The central panel (stick representation) illustrates the interactions between delafloxacin, S79 (ParC), D435 (ParE), a Mg²⁺ cation and selected nitrogenous bases of the DNA strand represented as a discontinuous white line. A weak hydrophobic interaction is seen between serine 81 of GyrA and the ring at position 2, along with two hydrogen bonds with the nitrogenous bases (thymine and cytosine) of the DNA double strand.

Figure 5 shows the comparison of levofloxacin and delafloxacin complexes with gyrase (left) and topoisomerase (right). On the right side, the carboxylic acids at position 3 of both quinolones are oriented in the same direction and interact via hydrogen bonds with the side chain of S79 in ParC. However, one of the most notable differences is observed in the topoisomerase complexes, such as the formation of a hydrogen bond between D435 of the ParE topoisomerase subunit and the 6-amino-3,5-difluoro-2-pyridinyl substituent at position 1 of delafloxacin. Since the levofloxacin substituent at position 1 is less bulky than the delafloxacin substituent and cyclizes forming an ether on the oxygen at position 8 of levofloxacin, this substituent is not able to interact and provides additional stability. Regarding delafloxacin, its interactions with gyrase (Figure 5, left) are less numerous and appear weaker than those observed in the delafloxacin–topoisomerase heterodimer complex (right). The major differences between the gyrase–delafloxacin complex and the levofloxacin complex are based in the additional interactions formed between the delafloxacin substituent at position 1 and the pyrimidine bases of the DNA double strand.

## Discussion

In this study we analysed the relationship between mutations in the QRDR genes and the susceptibility to levofloxacin (third-generation fluoroquinolone) and delafloxacin (fifth-generation fluoroquinolone), and tried to understand the main differences in terms of cross-resistance of these two fluoroquinolones in a model of clinical *S. pneumoniae* isolates that were susceptible and with different levels of levofloxacin resistance. We found that there is not a new specific mutation for delafloxacin in comparison with those previously described that leads to levofloxacin resistance and no differences have been found regarding the QRDR mutations previously described in *S. pneumoniae* that lead to resistance to other fluoroquinolones like moxifloxacin, ciprofloxacin, gatifloxacin, grepafloxacin or trovafloxacin.^8,19–21^ We also confirmed the preserved efficacy of delafloxacin against levofloxacin-resistant strains (87% delafloxacin susceptible).

All isolates with levofloxacin MICs of ≤2 mg/L lacked mutations in *gyrA*, *gyrB*, *parC* and *parE* associated with resistance. Although some mutations such as *gyrB* R477G/C and *parC* K137N were identified in this group, these did not affect susceptibility, suggesting that both mutations represent polymorphisms without phenotypic impact, as previously described.^6,15^

Regarding the low-level levofloxacin-resistant isolates (MICs of 3–12 mg/L) we observed that a single mutation in *parC* or in *gyrA* generated low-level resistance to levofloxacin but not to delafloxacin (Table 5). In addition, a significant number of isolates in this group did not present QRDR mutations. This lack of genotype–phenotype correlation could suggest the involvement of other resistance mechanisms, such as efflux pumps or porin deficiency;^6^ however, these mechanisms are very infrequent and have only been described in isolates with low levels of resistance to fluoroquinolones.^22^

Among highly levofloxacin-resistant isolates, a clear pattern of mutation accumulation was observed, with *gyrA* being the most frequently affected gene. The S81F mutation was the most prevalent, followed by S81Y, S81L and their combinations with the *parC* mutation S79F. This pattern is consistent with previous reports,^9,14,15^ which describe the progressive acquisition of mutations in *gyrA*, followed by *parC* and eventually *parE*, as the main evolutionary mechanism leading to high fluoroquinolone resistance. The simultaneous presence of mutations in *gyrA* and *parC* was common in this group of isolates, and in some cases up to four resistance-associated mutations were detected. A total of 23.9% of isolates in this group (11/46) showed delafloxacin MICs of ≥0.12 mg/L, but 9 of these presented delafloxacin MICs of 0.094 mg/L, an MIC at the limit of the cut-off point established for resistance, indicating other possible mechanisms of resistance that do not affect the activity of delafloxacin. The elevated MICs were always associated with the accumulation of at least one mutation in *gyrA* concomitantly with at least one mutation in *parC* (Figure 5). In this group, serotype 8 isolates were the most frequent. This is important because in Spain a lineage of this serotype (CC53/GPSC3) has recently emerged in children and adults with high potential to cause invasive disease due to the presence of a very efficient PspC protein.^23^

Mutations within the QRDRs of Gram-positive bacteria are pivotal in the emergence of fluoroquinolone resistance, including resistance to delafloxacin. In three studies evaluating the *in vitro* activity of delafloxacin against *Staphylococcus aureus*,^24–26^ mutations in the genes encoding DNA gyrase and topoisomerase IV were reported as major contributors to resistance, *gyrA*S84L and *parCS*80F being the most frequently identified mutations. In our analysis of *S. pneumoniae*, the most prevalent mutations were *gyrA*S81 and *parC*S79, with phenylalanine being the most common replacement. These findings are consistent with the substitutions observed in *S. aureus*, particularly in the *parC* subunit.

Although serine residues are commonly found in protein-active centres due to their reactive hydroxyl group, which can form hydrogen bonds with polar substrates, the observed substitutions are relatively conservative in nature. These mutations appear to alter local hydrophobicity and structural stability within the enzyme’s active site, thereby compromising drug-binding affinity (Figure 5). Such substitutions were consistently identified in levofloxacin-resistant *S. pneumoniae* isolates, suggesting that they constitute a foundational mechanism of resistance to quinolones, as previously reported.^6,15^ Similar changes in the polarity of the amino acid substitutions have also been suggested as important factors contributing to resistance of Gram-negative microorganisms to delafloxacin.^27,28^

Interestingly, while most of these substitutions involved phenylalanine, a bulky non-polar residue, our cohort also included isolates with mutations to tyrosine, a similarly bulky but polar amino acid. Specifically, six isolates exhibited the S81Y mutation in *gyrA*, five presented the S79Y mutation, and four carried the D83Y mutation in *parC*. All of these were associated with levofloxacin resistance, while showing a variable, though generally susceptible, range of delafloxacin MICs (0.003–0.09 mg/L). These findings suggest that substitutions at QRDR positions, involving the loss of a highly reactive polar residue such as serine in favour of non-polar (phenylalanine) or bulky polar (tyrosine) residues, may disrupt the stability of the drug–target complex and play a critical role in mediating quinolone resistance.^29^ In summary, several mutations in the QRDR related to specific amino acids that were frequently found in our isolates (*parC*S79, *gyrA*S81, *parE*D435) are very destabilizing because they are directly involved in the binding of the fluoroquinolones.

A particularly significant finding was the activity of delafloxacin against many of the highly levofloxacin-resistant strains. The acquisition of individual point mutations in *gyrA*, *parC* or *parE* did not significantly impact its activity, and the corresponding MIC values remained largely unaffected. In fact, of the total isolates with levofloxacin MICs of >32 mg/L, 76.1% remained delafloxacin susceptible. Even among those with three to four QRDR mutations, significant delafloxacin activity was observed. This can probably be explained by the delafloxacin interactions being more optimized in the active centre of both enzymes, which gives it activity even in the presence of mutations. However, the most critical factor is the accumulation of mutations, particularly when these occur concurrently in both target enzymes (Figure 3). In fact, we identified 11 isolates with cross-resistance to delafloxacin (MIC ≥ 0.12 mg/L), all of them with simultaneous mutations in *gyrA* and *parC.* This suggests that, although delafloxacin presents a higher genetic barrier to the development of resistance, it is not absolute, and the accumulation of specific mutations may eventually compromise its efficacy.

Another notable finding is the *parE*D435N mutation, which may directly affect protein structure through the loss of a hydrogen bond. Although this mutation is generally associated with the presence of prior resistance-conferring mutations, it also appears to contribute to high-level quinolone resistance, including resistance to delafloxacin. Of the 12 isolates carrying this mutation, 3 (25%) were resistant to delafloxacin, with MIC values ranging from 0.12 to 0.5 mg/L. Despite this, the differential pharmacodynamic properties of delafloxacin, including its anionic structure, greater intracellular penetration, advantages in acidic environments and in biofilms, differences in the binding mode of delafloxacin to target enzymes, and a PTA of >90% at an MIC_90_ of 0.25 mg/L with the standard dosage approved (300 mg, q12h, IV), suggest a bactericidal effect even with MIC values above the EUCAST ECOFF.^30^

Finally, a high prevalence of the *parE*I460V mutation was detected in all isolates, with no apparent correlation with resistance, indicating that it is probably a neutral polymorphism widely distributed in the isolates analysed.

Our series includes a wide variety of pneumococcal serotypes (Table 3). A total of 23 isolates belonged to serotypes not included in the 20-valent conjugate vaccine. Although 21 of them were susceptible to delafloxacin, it is noteworthy that two isolates (serotype 15A) were resistant to both levofloxacin and delafloxacin (MIC ≥ 0.12 mg/L), highlighting the need for continued surveillance to detect resistant isolates. Serotype 4 was the most frequent, presenting low-level levofloxacin resistance. This is important from the epidemiological perspective, since this serotype has increased in recent years among young adults and is also affecting the elderly population.^31,32^

Some limitations of our study are that the characterization of the analysed strains was only phenotypic (serotype) but not genotypic [ST or clonal complex (CC)], and the lack of evaluation of other resistance mechanisms such as overexpression of efflux pumps. Although, infrequently, three different fluoroquinolone-efflux pumps have been described in pneumococci (PmrA, DinF and PatAB), PatAB is the only shown to confer low-level resistance to ciprofloxacin (8 mg/L) and levofloxacin (4 mg/L) in clinical isolates, with a low impact against other fluoroquinolones.^6,22,33^ In our study, 23 strains with low-level levofloxacin resistance did not present mutations in the QRDR, suggesting that resistance could be caused by efflux pumps; however, this possible mechanism did not affect the activity of delafloxacin. Therefore, our results do not seem to indicate that this mechanism has a main or relative role in the resistance of delafloxacin.

Another limitation is that the vaccination status of the patients was unknown; however, most cases corresponded to adults who were very probably not immunized with pneumococcal conjugate vaccines (PCVs). Vaccination with the 23-valent pneumococcal polysaccharide vaccine was recommended in Spain in 2001 for individuals aged >2 years at high risk of pneumococcal disease, and from 2005 its use was extended to adults aged >59 years. PCV13 was implemented in the Spanish national immunization childhood calendar in 2016. In our study, most of the highly levofloxacin-resistant strains were associated with serotypes related to conjugate vaccines: serotype 8 (PCV20 and PCV21), serotypes 9V and 14 (PCV13, PCV15 and PCV20) and serotype 15A (PCV21).

Finally, another limitation of this study is that we did not perform molecular typing of the isolates. However, most of the strains analysed belonged to a small number of serotypes (8, 9V, 15A and 14). Previous studies in Spain have shown that fluoroquinolone-resistant strains of serotypes 8 and 15A belong to CC63, and fluoroquinolone-resistant strains of serotype 9V belong to CC156.^34^ In particular, the emergence of levofloxacin-resistant serotype 8 ST63 (Sweden^15A^-25 clone) and serotype 9V ST156 (Spain^9V^-3 clone) occurred in Madrid some years ago.^35^ In addition, the existence of the penicillin- and erythromycin-resistant serotype 14,^36^ and the clonal spread of the serotype 14 variant of the Spain^9V^-3 clone in our country are well known.^37^

In summary, our results demonstrate that the presence of mutations in *gyrA* and *parC*, and even a single mutation in *gyrA*, strongly correlate with resistance to levofloxacin in *S. pneumoniae*, with *gyrA* as the initial key gene, followed by *parC* and *parE*, the mutations *gyrA*S81F/Y, *parC*S79F/Y and *parE*D435N being particularly relevant. However, delafloxacin requires the accumulation of multiple mutations to acquire resistance and maintained its activity against 76.1% of highly levofloxacin-resistant strains, including those with two or three QRDR mutations.

These findings support the value of delafloxacin as a highly active fluoroquinolone against *S. pneumoniae* in settings with a high prevalence of fluoroquinolone resistance, but also underscore the need for continued surveillance to detect potential emerging resistance events.^38^
